# Assessment of sexual function in postmenopausal breast cancer survivors

**DOI:** 10.1093/sexmed/qfae035

**Published:** 2024-06-28

**Authors:** Caroline Nakano Vitorino, Michelle Sako Omodei, Rafaela Caroline de Souza, Georgia Petri Nahas, Daniel de Araujo Brito Buttros, Eduardo Carvalho-Pessoa, Heloisa De Luca Vespoli, Eliana Aguiar Petri Nahas

**Affiliations:** Graduate Program in Obstetrics and Gynecology, Medical School, São Paulo State University, Botucatu, São Paulo 18618-970, Brazil; Graduate Program in Obstetrics and Gynecology, Medical School, São Paulo State University, Botucatu, São Paulo 18618-970, Brazil; Graduate Program in Obstetrics and Gynecology, Medical School, São Paulo State University, Botucatu, São Paulo 18618-970, Brazil; Department of Obstetrics and Gynecology, Medical School, São Paulo State University, Botucatu, São Paulo 18618-970, Brazil; Graduate Program in Obstetrics and Gynecology, Medical School, São Paulo State University, Botucatu, São Paulo 18618-970, Brazil; Graduate Program in Obstetrics and Gynecology, Medical School, São Paulo State University, Botucatu, São Paulo 18618-970, Brazil; Department of Obstetrics and Gynecology, Medical School, São Paulo State University, Botucatu, São Paulo 18618-970, Brazil; Graduate Program in Obstetrics and Gynecology, Medical School, São Paulo State University, Botucatu, São Paulo 18618-970, Brazil; Department of Obstetrics and Gynecology, Medical School, São Paulo State University, Botucatu, São Paulo 18618-970, Brazil; Graduate Program in Obstetrics and Gynecology, Medical School, São Paulo State University, Botucatu, São Paulo 18618-970, Brazil; Department of Obstetrics and Gynecology, Medical School, São Paulo State University, Botucatu, São Paulo 18618-970, Brazil

**Keywords:** breast cancer, sexual function, menopause

## Abstract

**Background:**

Breast cancer (BC) is considered a risk factor for sexual dysfunction, which may be associated with the diagnosis itself or with oncological treatments. However, sexual dysfunction often remains underdiagnosed and unaddressed among BC survivors.

**Aim:**

The study sought to evaluate the sexual function of postmenopausal BC survivors compared with postmenopausal women without BC.

**Methods:**

This case-control study included 178 postmenopausal BC survivors (stages I-III), 45 to 70 years of age, with amenorrhea for ≥12 months and sexually active. They were compared with 178 women without BC, matched (±2 years) for age and time since menopause in a 1:1 ratio. Sexual function was evaluated using the Female Sexual Function Index (FSFI), which consists of 6 domains (desire, arousal, lubrication, orgasm, satisfaction, and pain), with a total score ≤26.5 indicating risk of sexual dysfunction. Statistical analysis included Student’s *t* test, chi-square test, and logistic regression (odds ratio [OR]).

**Outcomes:**

Evaluation of sexual function in postmenopausal women treated for BC.

**Results:**

Postmenopausal BC survivors showed poorer sexual function in the desire domain (*P =* .002). No significant differences were observed between groups in the other FSFI domains and total score (*P >* .05). Postmenopausal BC survivors had a higher prevalence of risk of sexual dysfunction (64.6% with a total score ≤26.5) compared with the control group (51.6%) (*P =* .010). Adjusted risk analysis for age and time since menopause revealed a higher risk of sexual dysfunction in BC survivors compared with women without cancer (OR, 1.98; 95% confidence interval, 1.29-2.96; *P =* .007). Among BC survivors, the use of hormone therapy was associated with a higher risk of sexual dysfunction (OR, 3.46; 95% confidence interval, 1.59-7.51; *P =* .002).

**Clinical Implications:**

Postmenopausal BC survivors should be regularly assessed before and throughout treatment to enable the early detection and diagnosis of sexual dysfunction.

**Strength and Limitations:**

The main strength is that this study might contribute to a better understanding of sexual function in postmenopausal BC survivors compared with women without BC. The main limitation is that while the FSFI is a valid and reliable tool for the evaluation of female sexual function, it does not allow a comprehensive diagnosis of sexual dysfunction, as it is not applicable to partners.

**Conclusion:**

Compared with postmenopausal women without BC, postmenopausal BC survivors face a higher risk of sexual dysfunction, especially when treated with adjuvant hormone therapy.

## Introduction

Breast cancer (BC), the most prevalent cancer in women worldwide,^1^ is considered a risk factor for sexual dysfunction, which may be associated with the diagnosis itself or with oncological treatments.^2^^,^^3^ Although adjuvant BC treatments can clearly improve survival, they may lead to adverse effects that undermine quality of life for years after diagnosis and treatment.^4^^,^^5^ Sexual health is recognized as a crucial component of a woman’s well-being and a basic human right,^6^ yet sexual dysfunction poses significant challenges to social, mental, and physical health, particularly in cancer survivors.^2^^,^^5^^,^^7^

The etiology of female sexual dysfunction is multifactorial, involving both biological factors and psychosocial elements.^6^ Cancer has significant physical and psychological consequences, and BC survivors are more prone to sexual dysfunction compared with those without cancer.^8-11^ Sexual function worsens with advancing menopause status^6^ and the prevalence rates of sexual dysfunction in postmenopausal women without cancer can range from 40% to 55%,^6^ while the reported rates among postmenopausal women with BC reach up to 92%.^11^ In this systematic review, the results were heterogeneous and with a small number of participants in most studies, and only 1 case-control study (women with and without BC).^11^ In order to evaluate sexual function objectively, several instruments assessing its different elements have been developed, taking into account the subjective nature of sexuality. The Female Sexual Function Index (FSFI)^12^ is recognized for its validity and reliability in assessing sexuality-related concerns among both women with and without cancer and is currently one of the most widely utilized instruments for evaluating female sexual function.^10^^,^^13^ A meta-analysis based on FSFI showed that approximately 74% of postmenopausal women with BC experience some degree of female sexual dysfunction.^10^

Previous studies have shown that women value sexual function as an important component of overall health.^6^ Thus, a comprehensive approach to the care of women treated for BC should recognize sexual function as a crucial determinant of their quality of life. ^5^^,^^13^^,^^14^ However, estimates suggest that less than half of these women seek and/or receive medical care.^15^ For various reasons, women do not discuss their concerns with their doctors, and healthcare providers may lack the training, knowledge, or comfort level to engage in discussions about sexual health.^5^ As BC survivorship improves, it is essential to investigate perceived health and well-being and to identify factors that may affect sexual function in order to prioritize rehabilitation after BC treatment.^4^ Sexual dysfunction often remains underdiagnosed and unaddressed among BC survivors.^5^^,^^15^

Given the barriers faced by BC patients from diagnosis through treatment and the impact of the disease on sexual health, do women after BC treatment have a higher risk of sexual dysfunction when compared with women of the same age and time since menopause? The objective of this study was to evaluate sexual function in postmenopausal women treated for BC compared with postmenopausal women without BC. The study also aimed to gain insights into the impact of oncological treatments on sexual function.

## Methods

### Study design and sample selection

This is a case-control study of women who attended a Hospital of Clinics, Botucatu Medical School, São Paulo State University, between 2021 and 2022. Inclusion criteria for the study group were postmenopausal women between 45 and 70 years of age, with a histological diagnosis of BC (stages I-III) for at least 12 months, who had completed cancer treatment (surgery, chemotherapy or radiotherapy), and who were sexually active (having engaged in at least 1 heterosexual relationship in the past month). Women currently on adjuvant endocrine therapy were eligible to be in the study. The control group consisted of postmenopausal women without BC, between 45 and 70 years of age, who were selected from a database and matched (±2 years) by age and time since menopause in a 1:1 ratio. The same method was used to assess sexual function in women with and without BC. The exclusion criteria for both groups were a history of alcohol or drug abuse, diagnosis of severe depression, cognitive deficits or illiteracy, use of menopausal hormone therapy (systemic or vaginal), and not having sexual activity in the last 4 weeks. Written informed consent was obtained from all participants, and the study was approved by the Research Ethics Committee (CAAE: 57897722.3.0000.5411).

### Clinical data

Data collected included age, age at menarche, age at menopause, time since menopause, age at first sexual intercourse, parity, current smoking status, sexual activity (at least 1 heterosexual relationship in the past month), history of chronic diseases (hypertension, diabetes, depression), medication use, physical activity, and blood pressure. The following data were obtained for anthropometric assessment: weight, height, body mass index (BMI) (weight/height^2^), and waist circumference (WC). The World Health Organization’s 2002 criteria were used to classify patients based on BMI as follows: ≤24.9 kg/m^2^ normal weight, 25 to 29.9 kg/m^2^ overweight, and ≥30 kg/m^2^ obese. WC was measured as the smallest circumference between the last rib and the iliac crest, with the measurement taken during expiration. A WC >88 cm was considered increased.^16^

### Sexual function assessment

Eligible patients were asked to complete FSFI questionnaire in a private room. An FSFI version already translated into Portuguese and validated for use in Brazil was used.^17^ This self-administered questionnaire consists of 19 items that assess 6 domains of sexual response: desire, arousal, lubrication, orgasm, satisfaction, and pain/discomfort.^12^ Each domain was scored individually on a 0 to 5 scale, with higher scores indicating greater levels of sexual functioning on the respective item. For pain-related questions, a reverse scoring scale was used.^18^ The sum of each domain score was multiplied by a domain factor ratio to ensure comparability, and then subsequently summed to derive a total FSFI score.^12^ Higher scores indicate better functioning, and a total score ≤26.5 indicates a risk of sexual dysfunction.^12^ Additionally, all patients were asked to rank their relationship with their partner as “poor,” “satisfactory,” “good,” or “very good.” The sexual function of women with BC and in the control group was evaluated by the same medical team.

### Oncological data

The following data were obtained from medical records: histopathological diagnosis of BC, histological grade, hormonal receptor status (estrogen receptor, progesterone receptor), human epidermal growth factor receptor 2 status, tumor stage, and treatments received (type of surgery, radiotherapy, chemotherapy, and adjuvant hormone therapy). Lymph node involvement was assessed either after surgery or by evaluating the sentinel lymph node. Tumor diameter was obtained from the histopathological reports, and the histological grade was classified as grade I (well differentiated), II (moderately differentiated), or III (poorly differentiated).^19^ Tumors were classified according to the simplified classification of the National Comprehensive Cancer Network into 3 categories: (1) localized invasive BC (stage I-II, T3N1M0), (2) locally advanced inoperable invasive BC (stage III), and (3) metastatic disease (stage IV).^20^

### Statistical analyses

Sample size was determined based on the study conducted by Jing et al^10^ that reported a 73.4% prevalence of sexual dysfunction among women treated for BC. Assuming a significance level of 5%, and type I and type II errors of 5% and 90%, respectively, the minimum sample size was estimated as 176 women with BC. Tables were created to analyze the clinical variables and parameters assessed for postmenopausal women, divided into groups: with and without BC. Data were assessed for normal distribution using the Shapiro-Wilk test, and for homogeneity using Levene’s test. Descriptive analysis included absolute frequencies and relative frequencies for categorical variables, as well as mean ± SD for quantitative variables. The analysis encompassed the following: (1) clinical variables (age, parity, age at first sexual intercourse, age and duration of menopause, smoking status, BMI, WC) and (2) sexual function (desire, arousal, lubrication, orgasm, satisfaction, pain/discomfort domains, and total score).

Group comparisons were performed using the chi-square test or Fisher’s exact test (for expected values ≤5) for categorical variables, and the *t* test and gamma distribution (for asymmetric variables) for quantitative characteristics. Logistic regression analysis was conducted to estimate the odds ratios (ORs) and their respective 95% confidence intervals (CIs) for influential variables associated with risk of sexual dysfunction (FSFI total score ≤26.5). For the construction of the multiple model, significant variables and those with *P* values <.20 from the univariate analysis were considered. Multivariate analysis was performed by binary logistic regression, to examine the association between the presence of BC, clinical parameters, and oncological parameters and treatments (independent or explanatory variables) and the risk of sexual dysfunction (dependent variable or response) in postmenopausal women, adjusted for age and time since menopause (confounding variables). A level of significance of 5% or the corresponding *P* value was adopted in all tests. The analyses were performed using SAS 9.2 software (SAS Institute).

## Results

The age at BC diagnosis was 49.5 ± 6.3 years and BC follow-up time was 6.6 ± 3.6 years. The analysis of the anatomopathological characteristics of BC showed that 58.7% of women had tumors ≤2 cm, 89.97% had ductal tumors, and 50.3% had grade II tumors. Additionally, 56.5% were in stage 1, 59.85% had negative axillary lymph nodes, 77.2% were positive for estrogen receptor, 73.1% were positive for progesterone receptor, and 72.8% were negative for human epidermal growth factor receptor 2. Regarding treatment, 70.2% (n = 125 of 178) underwent breast-conserving surgery (quadrantectomy), 91.1% received radiotherapy, and 68.6% received neoadjuvant chemotherapy. A total of 76.9% had used adjuvant hormone therapy (42.1% tamoxifen and 34.8% aromatase inhibitor [AI]), of which 29.7% were currently using tamoxifen and 16.8% AI (data not shown).

The clinical and anthropometric characteristics of women with BC (n = 178), matched by age and time since menopause with women without BC (n = 178), are presented in Table 1. The groups investigated were homogeneous for most of the variables assessed. The only significant difference observed was in WC, with a higher mean value among women with BC compared with the control group (91.1 cm vs 86.7 cm; *P =* .0002) (Table 1).

**Table 1:** Comparison of clinical and anthropometric characteristics between postmenopausal women treated for breast cancer and postmenopausal women without breast cancer (control).

**Parameters**	**Breast cancer** **(n = 178)**	**Without breast cancer** **(n = 178)**	***P* value**
Age, y	55.6 ± 6.3	54.9 ± 5.7	.146^a^
Age at menarche, y	12.9 ± 1.7	12.5 ± 1.6	.108^a^
Age at first sexual intercourse, y	19.2 ± 4.8	18.9 ± 3.1	.304^a^
Age at menopause, y	47.5 ± 4.1	48.2 ± 3.6	.846^a^
Time since menopause, y	7.1 ± 5.5	6.6 ± 5.6	.258^b^
Parity, number of children	2.2 ± 1.2	2.4 ± 1.4	.059^b^
BMI, kg/m^2^	28.3 ± 4.5	27.6 ± 4.3	.130^a^
WC, cm	91.1 ± 11.6	86.7 ± 11.0	.0002^a, c^
SBP, mm Hg	120.3 ± 12.1	118.7 ± 11.6	.173^a^
DBP, mm Hg	76.8 ± 8.8	77.7 ± 7.6	.275^a^
Currently smoking	21 (11.8)	29 (16.3)	.050^d^
Physical activity	51 (28.7)	56 (31.5)	.452^d^
Arterial hypertension	75 (42.1)	71 (39.9)	.507^d^
Diabetes	22 (12.4)	19 (10.7)	.337^d^

Values expressed as mean ± SD or as n (%).

Abbreviations: BMI, body mass index; DBP, diastolic blood pressure; SBP, systolic blood pressure; WC, waist circumference.

^a^ *t* test. ^b^Gamma distribution. ^c^Significant difference (*P < .*05). ^d^Chi-square test.

Table 2, which compares FSFI domain scores between women with and without BC, shows that women with BC had poorer sexual function in the desire domain (*P =* .002). The remaining domain scores (arousal, lubrication, orgasm, and satisfaction) and total score did not significantly differ between groups (*P >* .05). A statistically significant difference was found in the percentage of women with risk of sexual dysfunction, with 64.6% of women with BC and 51.6% of women without BC having an FSFI score ≤26.5 (*P =* .010) (Table 2). A higher proportion of women in the BC group rated their relationship with their partner as “very good” compared with women in the control group, regardless of the presence or absence of sexual dysfunction (40.9% vs 23.9% and 57.1% vs 25.6%, respectively; *P <* .05) (Table 3).

**Table 2:** Comparison of domain scores and total score of sexual function between postmenopausal women treated for breast cancer and postmenopausal women without breast cancer (control).

**Sexual function domain (FSFI)**	**Breast cancer** **(n = 178)**	**Without breast cancer** **(n = 178)**	***P* value**
Desire	3.1 ± 1.3	3.8 ± 1.6	**.002** ^a, b^
Arousal	3.3 ± 1.4	3.6 ± 1.5	.588^a^
Lubrication	3.5 ± 1.5	3.9 ± 1.2	.096^a^
Orgasm	3.7 ± 1.2	4.0 ± 1.2	.390^a^
Satisfaction	4.2 ± 1.5	4.6 ± 1.3	.405^a^
Pain	4.1 ± 1.8	4.3 ± 1.8	.536^a^
Total score	21.9 ± 7.5	24.2 ± 6.9	.188^a^
Sexual dysfunction	115 (64.6)	92 (51.6)	.010^b, c^

Values expressed as mean ± SD or n (%).

Abbreviation: FSFI, Female Sexual Function Index.

^a^Student’s *t* test. ^c^Significant difference (*P < .*05). ^c^Chi-square test.

**Table 3:** Association between the presence of sexual dysfunction and relationship with partner among postmenopausal women treated for breast cancer and postmenopausal women without breast cancer (control).

	**With sexual dysfunction**			**Without sexual dysfunction**		
	**Breast cancer (n = 115)**	**Control (n = 92)**	***P* value**	**Breast cancer (n = 63)**	**Control (n = 86)**	***P* value**
**Relationship with partner**			.019^a^			<.0001^a^
Very good	47 (40.9)	22 (23.9)		36 (57.1)	22 (25.6)	
Good	48 (41.7)	49 (53.3)		25 (39.7)	47 (54.6)	
Satisfactory	17 (14.8)	17 (18.5)		2 (3.2)	16 (18.6)	
Poor	3 (2.6)	4 (4.3)		0 (0.0)	1 (1.2)	

Values are n (%).

^a^Significant difference (*P <* .05; chi-square or Fisher’s exact test).

Women with BC had a 2-fold higher risk of sexual dysfunction compared with those without cancer (OR, 1.98; 95% CI, 1.29-2.96; *P =* .007). A poor/satisfactory relationship with their partner was also associated with an increased risk of sexual dysfunction (OR, 1.78; 95% CI, 1.10-3.12; *P =* .046). Adjuvant hormone therapy use among women with BC was associated with a higher risk of sexual dysfunction (OR, 3.46; 95% CI, 1.59-7.51; *P =* .002). No significant associations were observed for the other variables analyzed (Table 4).

**Table 4:** Multivariate analysis adjusted for the presence of breast cancer, clinical parameters, and oncological treatments influencing the risk of sexual dysfunction (FSFI total score ≤26.5) in postmenopausal women.

**Variable**	**OR**	**95% CI**	***P* value**
**Overall (n = 356)**			
Breast cancer	1.98	1.29-2.96	.007^a^
Physical activity	0.84	0.56-1.32	.445
Smoking	1.56	0.76-3.21	.229
Obesity	1.01	0.94-1.11	.818
Waist >88 cm	0.99	0.96-1.02	.651
Poor relationship with partner	1.78	1.10-3.12	.047^a^
Breast cancer group (n = 174)			
Mastectomy	1.12	0.39-1.32	.827
Staging	1.63	0.78-3.41	.195
Chemotherapy	1.32	0.55-3.16	.533
Hormone therapy	3.46	1.59-7.51	.002^a^

The OR was adjusted for age and time since menopause. Stating: stage 1 × stages 2 + 3.

Abbreviations: CI, confidence interval; FSFI, Female Sexual Function Index; OR, odds ratio.

a Significant difference (*P <* .05; logistic regression).

## Discussion

This study showed that the incidence of risk for sexual dysfunction was higher in postmenopausal BC survivors compared with postmenopausal women without BC. Additionally, BC was identified as a risk factor for sexual dysfunction, particularly among women who received hormone therapy as part of their adjuvant treatment. Among the women with BC participating in this study, FSFI scores in the desire domain were lower compared with women without BC. However, scores in all other domains (arousal, lubrication, orgasm, satisfaction, and pain/discomfort) did not significantly differ between the groups.

These findings are in line with a recent review, which demonstrated persistent low sexual desire among BC survivors, from diagnosis to post-treatment.^13^ Female sexual dysfunction is a highly complex phenomenon and a well-described consequence of comprehensive BC care.^21^ In a prospective study of sexual function in 226 women before and after BC surgery, Cornell et al^22^ observed that in women who had total mastectomy experienced significant reductions in overall FSFI sexual function, and decline in all individual FSFI domains except desire, which remained unchanged.^22^ These results differ from our findings, possibly due to the age of their participants, which ranged from 28 to 79 years, whereas our study specifically focused on postmenopausal women. Sexual function is known to worsen with the onset of menopause, regardless of age,^6^ and hypoactive sexual desire is an issue of particular concern in the postmenopausal women population.^23^^,^^24^ During the postmenopausal period, sexual dysfunction affects over 50% of women,^6^^,^^24^ and most women with BC are in the postmenopausal stage.^25^

Our study found a 64.6% frequency of risk for sexual dysfunction in postmenopausal women with BC, a rate higher than that observed in women of the same age and time since menopause without BC. This result is consistent with previous uncontrolled observational studies, such as those by Gambardella et al,^26^ Yan et al,^27^ and Yuan et al,^28^ which reported sexual dysfunction rates of 69%, 75.4%, and 76.4%, respectively, among women with BC. Similarly, meta-analyses have reported a prevalence of sexual dysfunction in women with BC of 65.5%^9^ and 73.4%.^10^ Differences in cultural factors, age range, adjuvant treatments, and metrics used to assess sexual function may explain these variations. For instance, a study by Ooi et al,^29^ which evaluated 94 Malaysian women with BC 18 to 65 years of age, found a prevalence of sexual dysfunction of 73% over a 12-month follow-up period,^29^ likely influenced by the younger age range of the patients. Women with BC in the premenopausal stage experience the symptoms of adjuvant therapies more abruptly than postmenopausal women, with a greater impact on sexuality.^25^ A recent French population study demonstrated that among women with BC (36-57.5 years of age), 34% did not engage in sexual intercourse, 34% had dyspareunia, and 30% were not satisfied with their sex life. Therefore, efforts to detect sexual disorders in BC survivors and offer quality support must be pursued.^30^

Our results showed that the risk of sexual dysfunction was approximately twice as high in postmenopausal women with BC compared with women without BC (OR, 1.98; 95% CI, 1.29-2.96), especially among those using hormone therapy as part of adjuvant cancer treatment (OR, 3.46; 95% CI, 1.59-7.51). In a cross-sectional study by Fogh et al,^31^ which involved 227 women treated for BC ≥18 years of age, 85% of the patients reported experiencing worsened sexual life after BC, with 68.9% reporting reduced sexual desire, and 58% attributing sexual dysfunction to cancer treatment.^31^ Sexual function impairments are common among BC survivors, especially among those undergoing adjuvant endocrine therapy.^5^^,^^31^ In a population-based study, BC survivors were at higher risk of sexual dysfunction diagnosis compared with the general population. This risk increased 2.05-fold within 1 to 5 years after cancer diagnosis (95% CI, 1.89-2.22) and 3.05-fold in women diagnosed with cancer at <50 years of age (95% CI, 2.65-3.51). Cancer treatments including endocrine therapy were associated with an increased risk of sexual dysfunction among BC survivors.^32^ A recent review, encompassing 37 studies investigating the link between BC and treatment-induced effects on sexual desire, concluded that surgical treatment and adjuvant hormonal therapy are significant factors influencing low sexual desire in BC.^13^

In our study, approximately 78% of women had hormone receptor–positive tumors, and 77% were using hormone therapy. Although current guidelines recommend up to 10 years of treatment,^33^^,^^34^ women with hormone receptor–positive BC typically receive adjuvant hormone therapy for at least 5 years to lower the risk of recurrence and mortality. However, estrogen deprivation is widely known to negatively affect sexual function.^35^ Adjuvant endocrine therapy in BC suppresses circulating estrogen levels and involves AIs and tamoxifen (a selective estrogen receptor modulator).^34^^,^^36^ These medications can trigger the onset of genitourinary syndrome of menopause symptoms or worsen existing symptoms.^37^^,^^38^ Menopause triggered by cancer treatment is typically abrupt, with more intense and prolonged estrogen deprivation symptoms, often resulting in more chronic side effects to vaginal health and surrounding tissues than with gradual estrogenic decline of menopause.^39^ AIs inhibit the aromatase enzyme that promotes the peripheral conversion of androgens to estrogens,^37^ leading to significantly higher rates of vaginal dryness and dyspareunia compared with tamoxifen.^38^ This might be explained by some estrogenic effect of tamoxifen on vaginal tissues in postmenopausal women, while AIs drastically reduce plasma estradiol levels.^21^ In a recent study, 47.6% of 168 women with BC were reported to be sexually inactive, regardless of AI use. However, those on AI therapy had significantly higher risk of sexual dysfunction and worse quality of life.^21^ Another population-based Norwegian study among long-term BC survivors revealed that 52% of women were sexually inactive, with lack of interest being the most common reason. Among sexually active women, the same study showed that poor body image, chemotherapy, and current endocrine treatment were associated with reduced sexual activity.^40^ Although genitourinary syndromes of menopause symptoms are debilitating and painful for sexual intercourse, having cancer is also associated with problems in sexual function, such as decreased sexual desire,^41^ as observed in our study.

In our study, women with BC more frequently perceived their relationship as very good compared with women without BC, regardless of the presence of sexual dysfunction. However, a poor or unsatisfactory relationship with their partner was associated with a higher risk of sexual dysfunction (OR, 1.78; 95% CI, 1.10-3.12). The diagnosis of BC itself, aspects of personality and previous personal and sexual relationships influence the sexual function of patients with BC.^21^ In contrast to our study, which included only sexually active women with current partners, Cornell et al^22^ evaluated 226 American women with BC who were married, single, divorced, or widowed but did not observe any association between sexual dysfunction and marital status.^22^ On the other hand, in a study of factors associated with sexual morbidity in 378 French women treated for BC, Brédart et al^42^ observed that the absence of sexual activity or sexual dissatisfaction was associated with feelings of emotional detachment between the couple or fear of the sexual relationship with the partner. Additionally, women with poorer marital relationships experienced challenges in sexual satisfaction. The authors concluded that the perception of the couple’s relationship played a significant role in the experience of sexual problems among women with BC.^42^

Sexual dysfunction can negatively impact relationships, self-esteem, and overall quality of life. However, it is still poorly addressed in clinical practice.^5^^,^^24^ This study might contribute to a better understanding of sexual function in postmenopausal BC survivors compared with women without BC. Nevertheless, some limitations should be acknowledged. First, the study design used does not allow establishing a cause-and-effect relationship, and other uncontrolled variables might have influenced sexual function among study participants. Second, despite reaching the required number of study participants, sample size was relatively limited. Third, while the FSFI is a valid and reliable^12^ tool for the evaluation of female sexual function, it does not allow a comprehensive diagnosis of sexual dysfunction, as it is not applicable to partners. Moreover, it does not assess homosexual patients and/or nonpenetrative sexual practices. Fourth, the pre-existing sexual function of postmenopausal BC patients was unknown. Fifth, the FSFI focuses on vaginal intercourse and does not comprehend the involvement of the breasts in the sexuality. Extragenital erogenous zone stimulation during foreplay is important for women to reach orgasm. Younis et al,^43^ in a cross-sectional cohort study with 150 women with regular sexual activity and who responded to a self-administered questionnaire, observed that extragenital erogenous zones were found in 95.3% of women. In a descending order, the most powerful erogenous zones were breasts and nipples (84.6%), lips, neck, ears, and buttocks.^43^ Finally, even after excluding women with diagnosed depression or undergoing depression treatment, a number of patients may have presented undiagnosed anxiety or depression symptoms that could interfere with sexual function.

## Conclusions

Compared with postmenopausal women without BC, postmenopausal BC survivors face a higher risk of sexual dysfunction, especially when treated with adjuvant hormone therapy. Women with BC should be regularly assessed before and throughout treatment to enable the early detection and diagnosis of sexual dysfunction. Addressing sexual function in routine evaluations and providing follow-up and interventions to improve any possible sexual dysfunction are essential to enhance the overall quality of life for women with BC.

## References

[1] Sung H , Ferlay J, Siegel RL, et al. Global cancer statistics 2020: GLOBOCAN estimates of incidence and mortality worldwide for 36 cancers in 185 countries. CA Cancer J Clin. 2021;71(3):209–249. 10.3322/caac.21660 33538338

[2] 2-Candy B, Jones L, Vickerstaff V, et al. Interventions for sexual dysfunction following treatments for cancer in women. Cochrane Database Syst Rev 2016;2():CD005540. 10.1002/14651858.CD005540.pub3. 26830050 PMC9301918

[3] Pinto MAO , Calafiore D, Piccirillo MC, Costa M, Taskiran OO, de Sire A. Breast cancer survivorship: the role of rehabilitation according to the international classification of functioning disability and health-a scoping review. Curr Oncol Rep. 2022;24(9):1163–1175. 10.1007/s11912-022-01262-8 35403973 PMC9467947

[4] Roine E , Sintonen H, Kellokumpu-Lehtinen PL, et al. Health-related quality of life of breast cancer survivors attending an exercise intervention study: a five-year follow-up. In Vivo. 2020;34(2):667–674. 10.21873/invivo.11821 32111767 PMC7157873

[5] Hernández-Blanquisett A , Quintero-Carreño V, Álvarez-Londoño A, Martínez-Ávila MC, Diaz-Cáceres R. Sexual dysfunction as a challenge in treated breast cancer: in- depth analysis and risk assessment to improve individual outcomes. Front Oncol. 2022;12:955057. 10.3389/fonc.2022.955057 35982958 PMC9378851

[6] Scavello I , Maseroli E, Di Stasi V, Vignozzi L. Sexual health in menopause. Medicina (Kaunas). 2019;55(9):559. 10.3390/medicina55090559 31480774 PMC6780739

[7] Carter J , Lacchetti C, Andersen BL, et al. Interventions to address sexual problems in people with cancer: American Society of Clinical Oncology clinical practice guideline adaptation of Cancer Care Ontario guideline. J Clin Oncol. 2018;36(5):492–511. 10.1200/JCO.2017.75.8995 29227723

[8] Rodrigues-Machado N , Quintana MJ, Gómez-Gómez R, Bonfill-Cosp X. Sexual function in women with breast cancer: an evidence map of observational studies. Int J Environ Res Public Health. 2022;19(21):13976. 10.3390/ijerph192113976 36360854 PMC9654538

[9] Maiorino MI , Chiodini P, Bellastella G, Giugliano D, Esposito K. Sexual dysfunction in women with cancer: a systematic review with meta-analysis of studies using the female sexual function index. Endocrine. 2016;54(2):329–341. 10.1007/s12020-015-0812-6 26643312

[10] Jing L , Zhang C, Li W, Jin F, Wang A. Incidence and severity of sexual dysfunction among women with breast cancer: a meta-analysis based on female sexual function index. Support Care Cancer. 2019;27(4):1171–1180. 10.1007/s00520-019-04667-7 30712099

[11] Esmat Hosseini S , Ilkhani M, Rohani C, Nikbakht Nasrabadi A, Ghanei Gheshlagh R, Moini A. Prevalence of sexual dysfunction in women with cancer: a systematic review and meta-analysis. Int J Reprod Biomed. 2022;20(1):1–12. 10.18502/ijrm.v20i1.10403 35308323 PMC8902793

[12] Rosen R , Brown C, Heiman J, et al. The female sexual function index (FSFI): a multidimensional self-report instrument for the assessment of female sexual function. J Sex Marital Ther. 2000;26(2):191–208. 10.1080/009262300278597 10782451

[13] Luo F , Link M, Grabenhorst C, Lynn B. Low sexual desire in breast cancer survivors and patients: a review. Sex Med Rev. 2022;10(3):367–375. 10.1016/j.sxmr.2022.02.001 35410784

[14] Martins R , Otero P, Torres ÁJ, Vázquez FL. Quality of life and sexual satisfaction in women with breast cancer undergoing a surgical treatment and in their male partners. J Clin Med. 2022;11(23):6960. 10.3390/jcm11236960 36498539 PMC9739064

[15] Boswell EN , Dizon DS. Breast cancer and sexual function. Translational andrology and urology. 2015;4(2):160–168. 10.3978/j.issn.2223-4683.2014.12.04 26816822 PMC4708123

[16] NCEP . Executive summary of the third report of the National Cholesterol Education Program (NCEP) expert panel on detection, evaluation, and treatment of high blood cholesterol in adults (adult treatment panel III). JAMA. 2001;285(19):2486–2497. 10.1001/jama.285.19.2486 11368702

[17] Thiel R , Dambros M, Palma PC, Thiel M, Riccetto CLZ, Ramos MF. Translation into Portuguese, cross-national adaptation and validation of the female sexual function index. Rev Bras Ginecol Obstet. 2008;30(10):504–510. 10.1590/S0100-72032008001000005 19082387

[18] Pacagnella RC , Martinez EZ, Vieira EM. Construct validity of a Portuguese version of the female sexual function index. Cad Saude Publica. 2009;25(11):2333–2344. 10.1590/S0102-311X2009001100004 19936472

[19] Elston EW , Ellis IO. Method for grading breast cancer. J Clin Pathol. 1993;46(2):189–190. 10.1136/jcp.46.2.189-b PMC501162 8459046

[20] Gradishar WJ , Anderson BO, Balassanian R, et al. Invasive breast cancer version 1.2016, NCCN clinical practice guidelines in oncology. J Natl Compr Cancer Netw. 2015;13(12):1475–1485. 10.6004/jnccn.2015.0176 26656517

[21] Lubián López DM , Butrón Hinojo CA, Sánchez-Prieto M, Mendoza N, Sánchez-Borrego R. Sexual dysfunction in postmenopausal women with breast cancer on adjuvant aromatase inhibitor therapy. Breast Care. 2021;16(4):376–382. 10.1159/000510079 34602943 PMC8436603

[22] Cornell LF , Mussallem DM, Gibson TC, Diehl NN, Bagaria SP, McLaughlin SA. Trends in sexual function after breast cancer surgery. Ann Surg Oncol. 2017;24(9):2526–2538. 10.1245/s10434-017-5894-3 28560595

[23] Omodei MS , Marques Gomes Delmanto LR, Carvalho-Pessoa E, Schmitt EB, Nahas GP, Petri Nahas EA. Association between pelvic floor muscle strength and sexual function in postmenopausal women. J Sex Med. 2019;16(12):1938–1946. 10.1016/j.jsxm.2019.09.014 31680007

[24] Parish SJ , Simon JA, Davis SR, et al. International Society for the Study of Women's sexual health clinical practice guideline for the use of systemic testosterone for hypoactive sexual desire disorder in women. J Sex Med. 2021;18(5):849–867. 10.1016/j.jsxm.2020.10.009 33814355

[25] Kuehn R , Casaubon J, Raker C, Edmonson D, Stuckey A, Gass J. Sexual dysfunction in survivorship; the impact of menopause and endocrine therapy. Ann Surg Oncol. 2019;26(10):3159–3165. 10.1245/s10434-019-07552-z 31342358

[26] Gambardella A , Esposito D, Accardo G, et al. Sexual function and sex hormones in breast cancer patients. Endocrine. 2018;60(3):510–515. 10.1007/s12020-017-1470-7 29138989

[27] Yan R , Wang J, Yu J. Association of sexual attitudes with sexual dysfunction and sexual distress among Chinese breast cancer survivors: a cross-sectional study. Support Care Cancer. 2023;31(3):154. 10.1007/s00520-023-07600-1 36757498

[28] Yuan X , Wang J, Bender CM, Zhang N, Yuan C. Patterns of sexual health in patients with breast cancer in China: a latent class analysis. Support Care Cancer. 2020;28(11):5147–5156. 10.1007/s00520-020-05332-0 32060704

[29] Ooi PS , Draman N, Muhamad R, et al. Sexual dysfunction among women with breast cancer in the Northeastern part of West Malaysia. Sex Med. 2021;9(3):100351. 10.1016/j.esxm.2021.100351 34030061 PMC8240344

[30] Mangiardi-Veltin M , Mullaert J, Coeuret-Pellicer M, et al. Prevalence of sexual dysfunction after breast cancer compared with controls, a study from CONSTANCES cohort. J Cancer Surviv. Published June 6, 2023. 10.1007/s11764-023-01407-z 37278872

[31] Fogh M , Højgaard A, Rotbøl CB, Jensen AB. The majority of Danish breast cancer survivors on adjuvant endocrine therapy have clinically relevant sexual dysfunction: a cross-sectional study. Acta Oncol. 2021;60(1):61–68. 10.1080/0284186X.2020.1813326 32869712

[32] Chang CP , Ho TF, Snyder J, et al. Breast cancer survivorship and sexual dysfunction: a population-based cohort study. Breast Cancer Res Treat. 2023;200(1):103–113. 10.1007/s10549-023-06953-9 37160510 PMC10382144

[33] Pan H , Gray R, Braybrooke J, et al. 20-year risks of breast-cancer recurrence after stopping endocrine therapy at 5 years. N Engl J Med. 2017;377(19):1836–1846. 10.1056/NEJMoa1701830 29117498 PMC5734609

[34] Burstein HJ , Temin S, Anderson H, et al. Adjuvant endocrine therapy for women with hormone receptor-positive breast cancer: American society of clinical oncology clinical practice guideline focused update. J Clin Oncol. 2014;32(21):2255–2269. 10.1200/JCO.2013.54.2258 24868023 PMC4876310

[35] Streicher L , Simon JA. Sexual function post-breast cancer. Cancer Treat Res. 2018;173:167–189. 10.1007/978-3-319-70197-4_11 29349764

[36] Francis PA , Regan MM, Fleming GF, et al. SOFT Investigators; International Breast Cancer Study Group. Adjuvant ovarian suppression in premenopausal breast cancer. N Engl J Med. 2015;372(5):436–446. 10.1056/NEJMoa1412379 25495490 PMC4341825

[37] Sussman TA , Kruse ML, Thacker HL, Abraham J. Managing genitourinary syndrome of menopause in breast cancer survivors receiving endocrine therapy. J Oncol Pract. 2019;15(7):363–370. 10.1200/JOP.18.00710 31291563

[38] Lubián López DM . Management of genitourinary syndrome of menopause in breast cancer survivors: an update. World J Clin Oncol. 2022;13(2):71–100. 10.5306/wjco.v13.i2.71 35316932 PMC8894268

[39] Carter J , Baser RE, Goldfrank DJ, et al. A single-arm, prospective trial investigating the effectiveness of a non-hormonal vaginal moisturizer containing hyaluronic acid in postmenopausal cancer survivors. Support Care Cancer. 2021;29(1):311–322. 10.1007/s00520-020-05472-3 32358778 PMC7606252

[40] Smedsland SK , Vandraas KF, Bøhn SK, et al. Sexual activity and functioning in long-term breast cancer survivors; exploring associated factors in a nationwide survey. Breast Cancer Res Treat. 2022;193(1):139–149. 10.1007/s10549-022-06544-0 35226237 PMC8993724

[41] Sánchez-Borrego R . Sex after breast and gynecological cancer. Maturitas. 2015;81(1):106–107. 10.1016/j.maturitas.2015.02.018

[42] Brédart A , Dolbeault S, Savignoni A, et al. Prevalence and associated factors of sexual problems after early-stage breast cancer treatment: results of a French exploratory survey. Psychooncology. 2011;20(8):841–850. 10.1002/pon.1789 20568085

[43] Younis I , Fattah M, Maamoun M. Female hot spots: extragenital erogenous zones. Human Androl. 2016;6(1):20–26. 10.1097/01.XHA.0000481142.54302.08

